# Letter from W. W. Potter, Chairman, Prize Essay Committee

**Published:** 1885-08

**Authors:** W. W. Potter

**Affiliations:** Chairman, Prize Essay Committee, 306 Franklin street; Buffalo, N. Y.


BUFFALO, N. Y., July 23, 1885.

*To the Editor of the Buffalo Medical and Surgical Journal:*

For the information of all interested, the Committee on Prize Essays of the Medical Society, of the State of New York, desires to state, that the Society offers a prize of one hundred dollars, payable from the Merritt H. Cash prize fund, for the best original essay on any medical or surgical subject.

The conditions are: That the competitors shall reside in the State of New York; that the essays shall be either printed or type-written; that each essay shall be designated by a motto on the title page; that a corresponding motto, together with the name of the writer, shall be enclosed in a sealed envelope and attached to the essay; and that all essays shall be sent to the undersigned prior to January 1, 1886.

W. W. POTTER,
Chairman, Prize Essay Committee,
306 Franklin street.

